# Human Rights as Justifications in Wildernesses: A Third World Approach to EU Law

**DOI:** 10.1007/s42439-026-00119-z

**Published:** 2026-05-04

**Authors:** Aravind Ganesh

**Affiliations:** Faculty of Law, Politics and Sociology, University of Sussex, Falmer, UK

**Keywords:** EU External Relations, Grotius, Law of the Sea, Maritime Black Holes, Extraterritorial Jurisdiction, Western Sahara, Imperialism, Civilising Missions

## Abstract

This article makes three claims. First, that in his foundational writings, Hugo Grotius conceptualised the seas as institutional ‘wildernesses’ where public and private persons stand on equal footing. His purpose behind doing so was to advance the interests of European colonial companies. Second, while modern international law rejects Grotius’s fundamental juridical assumptions concerning the seas, it nevertheless retains the possibility of stretches of it reverting to wildernesses where juridical accountability is systematically foreclosed. Third, this foreclosure is exacerbated by, among other things, the European Union’s tendency to flit at will between an international legal person and an amorphous ‘union of values.’ This enables European Union officials to weaponise professed values of human rights and international law to advance distinctly neo-Grotian, neo-imperial agendas of resource extraction not just on the high seas, but in other ‘wildernesses’ like Non-Self-Governing Territories, whilst simultaneously avoiding juridical accountability.

This article makes three sequenced claims. First, in foundational writings on international law, Hugo Grotius conceptualised the seas as a ‘wilderness’; a deliberately maintained institutional void where public and private persons stood on equal footing. The overriding purpose behind his doing so was to justify the private, colonial wars of the Dutch East India Company against the Portuguese state for control of the South-East Asian spice trade.

Second, while the contemporary international law of the sea rejects Grotius’s empirical assumptions that the seas are unenclosable and inexhaustible, it retains his commitment to a liberal concept of freedom. As such, contemporary international lawyers might indeed ‘depart’ from Grotius, but only along paths determined by him. The upshot is that the modern law of the sea preserves the possibility of institutional voids emerging or being manufactured on stretches of maritime space. A core purpose of such ‘maritime black holes’ is the complete foreclosure of juridical accountability.

Third, several features of the European Union (EU) legal order exploit and exacerbate this problematic tendency. These include the restrictive rules on standing to bring judicial review of EU actions, but the more fundamental is the EU’s tendency to transmute arbitrarily from an international legal person into an amorphous ‘union of values,’ and back again. Altogether, these allow EU officials to weaponise professed values of human rights and fidelity to international law to advance distinctly neo-Grotian, neo-imperial agendas of resource extraction not just on the seas, but in other spaces characterisable as wildernesses, such as Non-Self-Governing Territories (NSGTs).

There is an alternative: a ‘Kantian’ conception of the seas advancing from a ‘republican’ conception of freedom as ‘cosmopolitan’ – and so, *inherently institutional* – thoroughfares through which the states of the world enter into ‘commerce’ with one another. Just as public roads in a domestic constitutional order cannot even be conceived without road safety acts, traffic regulations, etc., so too must the seas be conceived juridically as ‘global public goods’ always already governed by an institutional order. Such an account, it is argued, permits the assertion of extraterritorial jurisdiction from, and on condition of, internal and external judicial accountability including for human rights. For reasons of space, and because it is the subject of ongoing research, this alternative account cannot be given here. Instead, it is outlined in a ‘sister’ article setting out a Kantian account of the human right to rescue on the high seas. (Ganesh 2024) This article and the sister piece can be read independently of one another.

## The Fount of Sovereignty and the Geographic Scope of the EU Legal Order

Advocates General (A-Gs) of the Court of Justice of the European Union (CJEU) generally prefer the Court to follow their advice. Except for the occasional maverick desirous of establishing a reputation for intellectual independence, perhaps with an eye to a political career, it is a matter of professional embarrassment for the bench to reject an A-G’s Opinion. If so, then EU external relations law is a minefield: the law reports are littered with examples of the CJEU ignoring the A-G’s advice to rule differently.

For a case in point, consider *Air Transport Association* decision, where the CJEU was faced with the question of whether it was a violation of international law for the EU Emissions Trading Directive (2008) to impose carbon emissions offsetting requirements on airplanes overflying the high seas or the territory of foreign states. A-G Kokott advised the Court to invoke the ‘effects doctrine’ of extraterritorial jurisdiction; that is, the idea that ‘any State may impose liabilities, even upon persons not within its allegiance, for conduct outside its borders which has consequences within its borders which the State reprehends…’ (*Opinion of A-G Kokott*,* Air Transport Association*, 2011, para. 154; see also *United States v Aluminum Co of America*, 1945, p. 444).

Instead, the CJEU began its analysis with Article 3(5) TEU, which provides that ‘In its relations with the wider world, the Union shall uphold and promote its values and interests… It shall contribute to… the protection of human rights…, as well as to the strict observance and the development of international law, including respect for the principles of the United Nations Charter.’ It concluded from here that the ‘extraterritorial’ imposition of emissions offsetting requirements upon foreign airplanes overflying the high seas and the airspace of non-Member States was in perfect conformity with the EU’s obligation to ‘observe international law in its entirety, including customary international law, which is binding upon the institutions of the European Union.’ (*Air Transport Association of America*, 2011, para. 101) Such ‘extraterritorial’ carbon offsetting charges offended neither the customary rules of territorial jurisdiction nor the sovereignty of third states, because – so the court reasoned – they were collected only *after* the airplanes entered EU territory, at which point they became subject to the ‘*unlimited’* jurisdiction of the EU and the relevant Member State. (*Air Transport Association*, 2011, pp 124–126) (emphasis added).

In the wake of *Air Transport*, scholars celebrated the decision for effectively deploying Article 3(5) TEU as a ‘missionary principle’ justifying the projection of the EU’s exemplary values of environmental protection onto the wider world. (Broberg 2011; Herlin-Karnell 2014, pp 90–92) Others were more circumspect: a distinguished international lawyer commented at the time that while its results were commendable, the reasoning by which it got there was ‘not convincing.’ (Benvenisti 2014, pp 13–14) By treating the foreign airplanes’ presence within EU Member State territory as sufficient grounds for imposing duties upon them anywhere in the world, *Air Transport* effectively exploded the difference between what international lawyers call ‘prescriptive’ and ‘enforcement’ jurisdiction. Yet others were completely skeptical, asking by what right the EU presumed to arrogate to itself ‘the role of legislator, fee collector, and lastly exclusive beneficiary of the revenues [of its emissions trading scheme], for the sake of the entire world… [as] in a supposed *civitas mundi*.’ (Gattini 2012, pp 982–983).

*Air Transport* was not, however, without precedent. The CJEU had previously applied the exact same heterodox interpretation of the international law of jurisdiction in *Poulsen and Diva Navigation* (1992), a case concerning the application of EU fishing regulation in the high seas. Even the rejection of A-G Kokott’s advice for its own theory of extraterritorial jurisdiction was not unparalleled. In the seminal *Wood Pulp* decision, A-G Darmon had advised that the then European Community’s competition regulation be applied to a cartel operating outside it on the basis of the aforementioned effects doctrine. (*Opinion of A-G Darmon*,* Wood Pulp I*, 1988, paras. 27–30) In contrast, the CJEU held the application of EU competition controls justifiable despite all cartel coordination having taken place outside the EU simply because minor ‘price announcements’ had been made within the EC. (*Wood Pulp I*, 1988, paras. 11–18) Whether those price announcements had had any tangible effects on prices in the EU internal market was immaterial. The mere making of them on Member State territory was the sole ‘jurisdictional hook’ necessary for the projection of the EC’s legal order outside of it.

Of greatest interest for present purposes, however, is *Salemink*, a rare and strangely-ignored instance of an A-G persuading the court on a question of EU external relations law. The case involved a Dutchman who had moved his primary address to Spain while working on an oil rig in the continental shelf adjacent to the Netherlands. Upon returning to dry land, he found that this decision had disqualified him from certain Dutch invalidity benefits, and so, brought suit in the Dutch courts. The issue was whether and to what extent EU fundamental freedoms – in particular, the freedom of establishment – extended to spaces outside the territories of the Member States, say, to oil rigs located on the Dutch continental shelf. (*Salemink*, 2012)

Perhaps aware of his colleagues’ misfortunes, A-G Cruz Villalón hedged his bets by giving the CJEU a selection of theories to pick and choose from. The first was an ‘easy’ option; to follow established precedent holding that EU fundamental freedoms applied to work carried out outside Member State territory if it was sufficiently closely connected to work ordinarily carried out in Member States. The second, more ‘innovative’ option was to explore whether and to what extent the continental shelf counted as Member State territory, and so, to expound the scope of Member States’ obligations under EU law in that region. (*Opinion of A-G Cruz Villalón*,* Salemink*, 2011, paras. 38–39, 41)

Regarding the second option, A-G Cruz Villalón made the following fascinating claims, which deserve close attention:

44. As a physical space under the sovereignty of a State, *the concept of territory covers territorial space as such and also airspace and maritime space*. In all cases it consists of areas, recognised by international law, where each State exercises exclusive sovereignty, although these areas do not represent the full extent of the domain in which States can exercise their sovereign powers, since international law also recognises the existence of extraterritorial State powers.
45. Just as the area in which State sovereignty can be exercised does not necessarily coincide exactly with the extent of its territory, neither do the competences of a State which derive from sovereignty always exhibit the exclusivity and absolute nature which are characteristic of sovereign power. On the contrary, precisely as a result of the progressive legal regulation of the international community, *the exercise of sovereignty is subject to variations in intensity*,* becoming less pronounced as the connection between the area of exercise and the territorial base of the State becomes weaker.…*.
47. If State sovereignty over territorial sea is already restricted in the way I have indicated, the characteristic powers of a sovereign State diminish progressively the further one travels, so to speak, from ‘dry land’, such powers becoming reduced in the case of the continental shelf, as we shall go on to see in more detail, to a collection of ‘sovereign rights’ intended to be used for particular purposes, and diluted to the mere exercise of certain freedoms upon reaching the high seas, where any claim to sovereignty is quite simply invalid. (*Opinion of A-G Cruz Villalón*,* Salemink*, 2011, paras. 44–47) (emphasis added).

From these observations about the state of international law, A-G Cruz Villalón argued that because EU competences are conferred by the Member States, ‘EU law will apply to whatever extent the Member States exercise official authority in the areas of competence conferred on the Union’, subject to limitations specified in the Treaties. (*Opinion of A-G Cruz Villalón*,* Salemink*, 2011, paras. 54–55)

In contrast to their shabby treatment of his colleagues, the CJEU silently took A-G Cruz Villalón’s bait and ruled that ‘(s)ince a Member State has sovereignty over the continental shelf adjacent to it—albeit functional and limited sovereignty—work carried out on fixed or floating installations positioned on the continental shelf, in the context of the prospecting and/or exploitation of natural resources, is to be regarded as work carried out in the territory of that State for the purposes of applying EU law.’ (*Salemink*, 2012, para. 35).

In this way, the *Salemink* Judgment and more specifically the Opinion outline a striking vision of the geographic scope of the EU’s jurisdictional powers. This vision stands in stark – indeed polar – contrast to the classic *dicta* by Story J in *The Apollon*:

The laws of no nation can justly extend beyond its own territories, except so far as regards its own citizens. They can have no force to control the sovereignty or rights of any other nation, within its own jurisdiction. And, however general and comprehensive the phrases used in our municipal laws may be, they must always be restricted in construction, to places and persons, upon whom the Legislature have authority and jurisdiction. (1824, p. 370)

In *Appollon*, sovereignty, and consequently authority and jurisdiction, are in principle limited to territory; they obtain outside of it only in carefully specified exceptions. In *Salemink*, by contrast, sovereignty is imagined like a fountain – indeed, an oil well – gushing from the metropole and spreading everywhere, with the waves petering out the further away they travel from the metropole. It would envelop the entire world if only head pressure at the source could be increased.

## Empire, Civilising Missions, and Sincerity

The *Salemink* account of the EU legal order – as a thing fanning outward ‘like radio waves from a transmitter’ – will remind readers trained in political science of ‘dispositional’ theories of Empire. (Doyle 1986, p. 24) The original such theory was advanced by JA Hobson, who argued that the apogee of European imperialism in the late 19th to early 20th century arose because the weak consuming power of the working class in the metropolitan core forced owners of capital to expand ever further outward to find ever new markets. This then necessitated the extension of the metropole’s municipal legal order to protect newly-acquired property rights, which lead to imperial competition in the peripheries, and ultimately, to endless war. (Hobson 1902, p. 91) The obvious solution would have been to raise living standards among the working class in the imperialist core through redistributive policies, but Hobson’s famous answer as to one reason why this was never even considered was that capitalists ‘simply and instinctively attach… themselves [to] any strong, genuine elevated feeling which is of service, fan it and feed it until it assumes fervour, and utilise it for their ends.’ (Hobson 1902, p. 208) Importantly, this disguising of mercenary incentives was in no wise a crude exercise in naked hypocrisy. Rather – and this is what made civilising missions uniquely effective – was that those professing them did so *sincerely*:

it is quite likely that Earl Grey thinks that the Chartered Company which he directs is animated by a desire to improve the material and moral condition of the natives of Rhodesia… [and] Leopold, King of the Belgians, has claimed for his government of the Congo—‘Our only programme is that of the moral and material regeneration of the country.’ (Hobson 1902, p. 209).

In this way, civilizing missions were, in the words of doyen of ‘Third World Approaches to International Law’ (TWAIL),

‘central to the imperial project… The basic intellectual structure of the mission consisted of a constellation of related ideas whereby a certain entity was characterized as ‘barbaric’, ‘helpless’, immature, savage, violent or unproductive. In each case, European intervention was required in order to transform and even protect this aberrant entity… The project of transformation is never complete, however, as the non-European entity, because of its resistance or because of its posited inherently savage nature, proves itself to be incapable of change.’ (Anghie 2023, pp 28–29).

One would have thought such sentiments – particularly the final one – would no longer be admissible in the contemporary rules-based international order (if it still exists); at least not since post-war decolonization and the folding of the former colonies into the community of ‘civilised nations.’ A key question, then, is how such an evidently imperial conception of the geographical scope of the EU legal order could have been deemed perfectly consistent with the progressive legal regulation of the international community by the CJEU in *Salemink*.

A second curiosity, as evidenced by the Story J’s dicta, is that the *Salemink* conception of the geographical scope of jurisdiction was *entirely unknown* in the age of high imperialism. Contrary to the recent arguments by some TWAIL and liberal scholars, claims of expansive rights of jurisdiction are not imperial but ‘neo-imperial’ developments. (Chimni 2022; Criddle 2024) As another TWAIL stalwart has recently observed,

There was no reason for an imperial power to exercise extraterritoriality of this sort during colonialism as its fiat ran through its extensive empire… modern principles of jurisdiction had little to do with imperialism. It was the end of imperialism which made it necessary for rules of jurisdiction to be formulated.’ (Sornarajah 2023, pp 33–34).

Answers to these questions cannot be supplied from EU legal doctrine alone, nor even from a study of international legal doctrine. Rather, the tools needed are historical, moral, and conceptual. Indeed, we must go back very far to solve them; even to the 16th -century writings of the Dutch jurist Hugo Grotius. Revisiting these, however, will allow us to see the EU’s reliance upon values, human rights, and international law in a new light.

## Artificial Wildernesses: Grotius and the Sea

The following section argues that the CJEU’s neo-imperial conception of the geographic scope of the EU’s competences emerges out of the problematic response of 20th -century international First *and* Third World lawyers to Grotius’s original framework for the law of the seas. The core of that problem, is that although 20th -century international lawyers tried earnestly to depart from Grotius, they did so only on terms he defined.

Grotius begins, as one might expect, in the beginning: ‘GOD at the creation and again after the Deluge, gave to Mankind in general a Dominion over Things of this inferior World.’ (Grotius 1625, p. 420 (bk. 2, chs. 2, [2(1)]) In the chapter of *Laws of War and Peace* entitled ‘*Of Things which belong in common to all Men*’, he sets out an account taking place in historical time – albeit a highly idealized one stitched together from a dazzling array of philosophical, literary, and scriptural sources – starting from a picture of a primitive condition where human beings held all things in common, but which becomes unstable as they individually and collectively as societies assume a ‘*gigantick* Life’; that is, as their consumption increases they decline in virtue individually and their collectivities become ever more complex in structure. Through a series of compacts both explicit and tacit, as Grotius tells it, primitive humans began gradually removing things from the common stock and reducing them to exclusive possession. This ‘just so’ story of commodification concludes with the institutions of property and territory. (Grotius 1625, p. 428 423-427 (bk 2, ch 2, [3(1) 2(2)-5])

For two reasons, the commodification process stops short of the seas and the air. The first is a ‘moral Reason’: ‘the Sea is of so vast an Extent, that it is sufficient for all the Uses that Nations can draw from thence, either as to Water, Fishing, or Navigation.’ (Grotius 1625, p. 428 (bk 2, ch 2, [3(1)]) The second, is a ‘natural Reason’: ‘the taking of Possession obtains only in Things that are limited’ such as land rather than unbounded liquids or gases. (Grotius 1625, p. 430 (bk. 2, chs. 2, [3(2)]) Note that the inexhaustibility argument is not a claim about the physical attributes of the seas or the air, but a *normative* claim: from the *fact* that the seas are vast enough to provide for everyone’s needs, it follows that it is *immoral* (rather than merely inefficient) to exclude others. In the earlier *Free Seas*, Grotius declared – in what are possibly his best lines – that

If any in so great a sea should take empire and jurisdiction wholly to himself from the common use, yet nevertheless he should be accompted an ambitious seeker of excessive dominion; if any should forbid others to fish, he could not escape the brand of the brainsick covetousness. But he that doth also hinder navigation whereby he loseth nothing, what shall we conclude of him? (Grotius 1609, p. 33 (ch 5).

By so combining normative claims with empirical observations, Grotius effectively elaborated a conception of the seas as juridical ‘wildernesses’; that is, as the remnants of a primeval, institutionless^1^ void consciously preserved by civilization amidst artificial institutions of property and sovereignty. He gives this thought its most sublime expression in the *Free Seas*, where he describes the ocean as

unmeasurable and infinite, the parent of things bordering upon heaven, with whose perpetual moisture the ancients supposed not only fountains and rivers and seas, but also the clouds and the very stars themselves, in some sort to be maintained, which finally compassing the earth (this seat of mankind) by the reciprocal courses of tides can neither be kept back nor included and more truly possesseth than is possessed. (Grotius 1609, p. 32)

The upshot is that the seas are

in the number of those things which are not in merchandise and trading, that is to say, which cannot be made proper. Whence it followeth, if we speak properly, no part of the sea can be accompted in the territory of any people…(Grotius 1609, p. 30).

It is important to note that, for Grotius, there was nothing *directly in nature* compelling this position. Rather, as Yanagihara explains through the distinction between the ‘law of nature itself (*simpliciter*)’ and ‘the law of nature in relation to particular institutions (*pro certo rerum statu*)’, it ‘is a man-made institution (*institutum*), not natural reason, that prohibits the sea from being occupied lawfully.’ (Yanagihara 1993, pp 152–153; see also Grotius 1625, pp 156–157 (bk 1, ch 1, [10(7)−11(1)]).

The Grotian model thus conceived remained dominant for *centuries*. As late as 1893, an arbitral panel in the *Fur Seals* arbitration denied the United States any ‘right of protection… in the fur seals frequenting the islands of the United States in the Bering Sea, when such seals are found outside the ordinary three-mile limit’ in the face of a British claim of a ‘right to come and go upon the high sea without let or hindrance, and to take therefrom at will and pleasure the produce of the sea.’ (*Fur Seals Arbitration*, 1895, pp 10, 12) Story J’s *dicta* in *Apollon* are a perfect distillation of Grotius’s model of the geographical scope of sovereignty.

## Wilderness Lost: Overturning Grotius

Clearly, neither of Grotius’s assumptions still holds today. First, the seas are now recognized as eminently *enclosable*. Beginning in 1945, the United States declared it ‘just and reasonable’ for it to exercise ‘jurisdiction and control’ over the continental shelf as the geophysical extension of the US’s landmass. (*Truman Proclamation*, 1945) The culmination of this process was the introduction of a ‘Constitution for the Oceans’ in UNCLOS. (Koh 1983, p. xxxiii), and the emergence of the concept of ‘functional jurisdiction’ over the high seas – that is, of the exercise of sovereign jurisdiction over maritime zones delineated by the functions states perform there, for instance, the territorial sea, contiguous zone, exclusive economic zone, etc. (Gavouneli 2007; Chap. 2)

In *North Sea Continental Shelf* the International Court of Justice (ICJ) overturned the Grotian doctrine that no part of the seas can be accompted within the territory of any state, holding instead that certain

submarine areas… [i.e. the continental shelf] may be deemed to be actually part of the territory over which the coastal State already has dominion – in the sense that, although covered with water, they are a prolongation or continuation of that territory, an extension of it under the sea. (*North Sea Continental Shelf Cases*, 1969, para. 43)

It followed accordingly that

the rights of the coastal State in respect of the area of continental shelf that constitutes a natural prolongation of its land territory into and under the sea exist *ipso facto* and *ab initio*, by virtue of its sovereignty over the land, and as an extension of it in an exercise of sovereign rights for the purpose of exploring the seabed and exploiting its natural resources…(*North Sea Continental Shelf Cases*, 1969, para. 19).

A-G Cruz Villalón’s depiction of the spatial expanse of Member State sovereignty in *Salemink*, upon which the geographic scope of EU fundamental freedoms is clearly the product of this new dispensation. An important point – whose irony will become apparent in Sect. 5 – is that the major proponents behind the extension of sovereignty over sections of the seas, and their ‘positivization’ through international instruments setting out legal doctrines such as that of the Exclusive Economic Zone and the Common Heritage of Mankind, were actually Third World coastal states eager finally to take control of their mineral and fisheries resources. (Enyew 2022, pp 461–463)

Second, and more obviously, there is now absolutely no doubt that the seas are *exhaustible*. In ‘*Tragedy of the Commons*’, (Hardin 1968) – arguably the most widely-cited scientific article of the 20th century despite a complete lack of empirical research – Garrett Hardin specifically blamed the ‘shibboleth of the “freedom of the seas”’ in the context of explosive human population growth in the Global South for bringing ‘species after species of fish and whales closer to extinction.’ (Hardin 1968, p. 1245) Such considerations, combined with the enclosure of the seas described immediately above, motivated the ICJ to reverse *Fur Seals*:

States have an obligation to take full account of each other’s rights and of any fishery conservation measures, the necessity of which is shown to exist in those waters… the former laissez-faire treatment of the living resources of the high seas has been replaced by a recognition of a duty to have due regard to the rights of other States and the needs of conservation for the benefit of all. (*Fisheries Jurisdiction Case*, 1974, para. 72)

The upshot of these developments is that ‘the sea no longer is wholly common’; even the seabed and subsoil beyond the continental shelf have been ‘internationalised’ and placed under the jurisdiction of an International Seabed Authority. (Oude Elferink 2009, p. 163) Both limbs of the Grotian model having been overturned, the bottom would appear to have fallen out of the Grotian model. As such, even if Grotius did father the original law of the sea, he cannot be described as the father of the modern international law of the sea. (Oude Elferink 2009, p. 166)

## Wilderness Regained: Continuities with Grotius

Despite all this, Grotius continues to be fêted by contemporary international lawyers: stalwarts such as Eyal Benvenisti and Cedric Ryngaert invoke him to advance arguments for their theories of wide-ranging sovereign rights of extraterritorial and unilateral jurisdiction. Ryngaert claims that ‘In the Grotian tradition, reason, ethics, and the law of nature may demand that international legal action be taken beyond the express will of states, and that existing positive law should be reinterpreted or even modified to this effect.’ (Ryngaert 2020, p. 61) More to the point, in a celebrated article, Benvenisti proposes wide-ranging obligations of sovereigns as ‘trustees of humanity’ to accommodate foreign stakeholders’ interests where doing so would be harmless and costless – say, to supply life-saving drugs, allow compulsory licensing of pharmaceutical products, and share disease samples – from Grotius’s torch metaphor. (Benvenisti 2013, pp 324–25) These trusteeship principles not only obligate; they also empower ‘legislating for humanity’:

The trusteeship concept offers a clear endorsement to democracies that wish to unilaterally promote global welfare.… As trustees of humanity, national decision-makers must regard themselves as partaking in a collective effort to promote global welfare…. In fact, the sovereign-as-trustee concept even obliges states to pursue such policies. (Benvenisti 2014, p. 12).

The upshot of either ‘Grotian-inspired’ theory of extraterritorial jurisdiction is that state actors which are, for whatever reason, particularly well-placed to advance global or ‘common’ interests such as the protection of human rights are, for that very reason alone, *entitled* to exercise jurisdiction extraterritorially. Moreover, they are so entitled even against opposition by other states, so long as certain procedural safeguards are met. As Benvenisti puts it, ‘the fact that some states fail to cooperate should not hinder those who wish to act in pursuit of improving global standards, provided that they take into account the interests of others when devising policies…’(Benvenisti 2014, p. 12) Likewise, Ryngaert understands the *Air Transport* decision as proposing a ‘novel ground of jurisdiction that aims at the protection of global public goods that are insufficiently protected by international solutions…’ (De Baere and Ryngaert 2013, p. 401).

The historical Grotius would likely have been surprised and somewhat baffled to find his name being invoked to argue for the EU’s rights of unilateral jurisdiction to advance ‘global public goods’; a category that presumably includes marine biodiversity conservation and the suppression of international piracy. Grotius was adamant that sovereigns were prohibited from doing anything about the former, and was at the very least dubious about their prerogatives with respect to the latter. Both are discussed in a fascinating intellectual exchange between Grotius and one of his critics.

Recall our contemporary disagreement with Grotius on the exhaustibility of the seas. Although the possibility of entire species of creation disappearing completely was first established by Georges Cuvier only in 1796, it was abundantly clear even during Grotius’s lifetime that overfishing was severely depleting marine resources. William Welwod, a Scottish jurist and mathematician, complained in a 1613 critique of the *Free Seas* that ‘whereas aforetime the white fishes daily abounded even into all the shores on the eastern coast of Scotland, now forsooth by the near and daily approaching’ of large-scale Dutch fishing fleets ‘the shoals of fishes are broken and so far scattered away from our shores and coasts that no fish now can be found worthy of any pains and travails, to the impoverishing of all the sort of our home fishers and to the great damage of all the nation.’ (Welwod 2004, p. 74).

While other defences of the ‘closed seas’ are better known, Welwod’s pamphlet is of particular interest because Grotius responded to it in an unpublished, unfinished work discovered only centuries after his death. (Grotius 2004) After faulting Scottish fishermen for lacking the industry of Dutchmen, (Grotius 2004, p. 123) Grotius supplied a further argument: *no sovereign had standing to regulate fishing on the high seas*. At the core of his argument was the Stoic maxim *sub optimo omnia rex imperio possidet singuli dominio*. (Seneca 2011, p. 172 (bk. 7, Chaps. 6, [3])) From the thought that jurisdiction and ownership were two different things, Grotius argued that ‘the “law-making” (*νοµοθετική*) power comes from sovereignty, not from ownership,’ and so, that while a ‘prince [might] make law for the maritime actions of his subjects, judge these acts, even impose tribute, [as well as] do this for his allies, if this has been agreed to by treaty,’ (Grotius 2004, p. 128) this is the limit of what could be asserted by way of extraterritorial jurisdiction. Having searched ancient authorities, Grotius concludes that ‘I do not find laws or tributes imposed upon foreigners when acting on the sea.’ (Grotius 2004, p. 129).

Grotius then drove the positive argument for his case home with a singularly brilliant precedent: the famous story of Julius Caesar’s encounter with a band of pirates off the coast of Cilicia in modern Turkey. Having been taken hostage off the coast of Anatolia, Caesar negotiated his ransom *upwards*. The pirates then

… kept him prisoner for nearly forty days, to his intense anoyance… As soon as the stipulated fifty talents arrived and the pirates duly set him ashore, he raised a fleet and went after them. He had often smilingly sworn, while still in their power, that he would capture and crucify them, and this is exactly what he did. (Suetonius Tranquillus 1957, p. 12)

Crucially, he did all this entirely as *a private citizen*. Grotius comments that ‘Caesar would no more have dared this on the sea than in the province, indeed would have committed *lese majesty*, if the sea had been as much the territory of the Roman people as the province itself.’ (Grotius 2004, p. 129).

This absence of *public* jurisdiction on the seas is central to Grotius’s entire intellectual project; its overriding goal, after all, was to justify and defend the Dutch East India Company’s *private* wars against Portugal for access to the spice trade. Indeed, the specific incident that inspired the works Grotius is lauded for was effectively an act of piracy: the attack and plunder of a Portuguese carrack – the *Santa Catarina* – off the coast of Singapore in 1603, by a Dutch East India Company vessel captained by Grotius’s cousin, Jacob van Heemskerck. As such, contrary to the picture painted by Benvenisti and Ryngaert, the authentic Grotian position is that sovereigns have no particular ‘jurisdiction’ on the open seas, and that insofar as they act in that space, they do so as, and on equal footing with, private persons. Anyone and everyone – from private colonial companies to sovereign princes – has, by reason of their human capacity for reason, standing to pronounce and enforce their peculiar conceptions of the demands of natural law.

In contemporary scholarship, the theme of ‘maritime black holes’ has been revived by Itamar Mann. (Mann 2018) Citing the example of the Search and Rescue or ‘SAR’ Convention, which divides the high seas into Search and Rescue zones in which states parties are allocated the sole responsibility to come to the rescue of seafarers in distress, Mann asks what would happen if they left some maritime area unallocated, or if the state in charge of an SAR zone collapsed into disorder. The answer, is that that zone would revert to a Grotian wilderness. Accordinlgy, insofar as the concept of ‘functional’ jurisdiction relies upon states ‘enclosing’ a former uninstitutionalised global ‘commons’ into a multiplicity of regions by delineation and demarcation, ‘maritime black holes’ remain unavoidable. (Mann 2018, p. 366)

To his credit, Grotius would have foreseen all this: in the *Free Seas*, he argues that if exclusive possession could be established over portions of the sea simply by drawing ‘a certain imaginary line’, then the ‘geometricians should long since have taken away the earth from us and the astronomers heaven.’ (Grotius 1609, p. 34) In other words, Grotius predicted something like the obverse of the 19th -century ‘Scramble for Africa’. A scramble *away* from Africa effectively follows from the SAR Convention’s allocation of *responsibilities* through delimitation of the sea by imaginary lines.

This explains the recent decision of the European Court of Human Rights (ECtHR) in *SS v Italy*, where a 7-member bench unanimously dismissed an application by survivors of a Mediterranean ‘pullback’ operation. (*SS and others v Italy*, 2025) Although Italian coast guard officials were the first to receive the applicants’ distress signal, they ‘outsourced’ their rescue obligations arising under several international treaties to Libya, leading to some 20 deaths and torture of returnees. As such, a rule has arguably crystallized – at least in the broad ‘European’ legal order comprising the EU and the ECtHR – that states are under no obligation to rescue seafarers imperiled in SAR Zones of other states, not even if they are the first or only public officials to receive distress signals.

Whereas much of the post-decision commentary castigates the ECtHR’s theory of ‘human rights jurisdiction’ as the reason for ‘accountability gaps’ on the high seas, (Mann 2025; Spagnolo 2025) I contend that the opposite is true, and that the ECtHR’s extraterritoriality jurisprudence is fine in principle. Rather, it is the law of the sea which, because of its Grotian heritage, systematically allows for stretches of the seas to be construed as left outside all institutional order. Likewise, the recent executive order by President Trump instructing the United States’ National Oceanic and Atmospheric Administration (NOAA) to fast-track licences for seabed mining in the section of the high seas known as ‘the Area’ cannot be understood as a departure from Grotius. (“Unleashing America’s Offshore Critical Minerals and Resources,” 2025) As we saw from Grotius’s response to Welwod, limitless resource extraction is entirely consistent with his framework. It instead represents ‘a moment of legal continuity: a reaffirmation of the law’s longstanding capacity to render the ocean as a space of sacrifice.’ (Marcos 2025) The contemporary conception of the oceans as recently tamed wildernesses only inverts Grotius’s assumptions. Maritime legal black holes emerge because the modern law of the sea ultimately still operates within Grotius’s conceptual universe. (Ganesh 2025)

## Earl Grey in Luxembourg: Article 3(5) TEU as an Unrebuttable Presumption of Rectitude

So far, we have seen how the conception of the sea as an institutional void is central to the Grotian framework, as well as how and to what extent it is retained by contemporary international law to facilitate endless resource extraction and juridical unaccountability. We now turn to the final question of how the EU exacerbates these problems.

The most fundamental and deep-rooted reasons lie in the EU’s *sui generis* nature and the principle of conferred competences. As Ester Herlin-Karnell has noted, when

powers are outsourced, both the accountability and the democratic dimension are challenged… In the EU context, it is often unclear who the actual legislator is, as agencies and private actors are vested with quasi‑legislative powers. Key features of due process may therefore be endangered, as it is often not clear how an individual could be guaranteed the right to judicial review. (Herlin-Karnell 2024, p. 98)

Hallvard Sandven and Antoinette Scherz set out more precisely the mechanisms by which the European seas, paradigmatically the Mediterranean, are transformed into institutional voids. Their basic argument is that while border policy is a competence emphatically reserved to the Member States, the enforcement of the border policies of several Mediterranean Member States is now outsourced to Frontex, which is the EU’s first uniformed law enforcement agency. As such, ‘there is an asymmetry at the heart of the EU’s border regime… the EU wields significant power in border control whilst… having no competences over *immigration* policy.’ (Sandven and Scherz 2022, p. 686) As they explain,

governments keen to keep anti-immigration forces in their domestic constituencies at bay have everything to gain from delegating the enforcement of their border regime to an agent that cannot make them accept unwanted asylum seekers—even (and especially) when those asylum seekers have claims that are protected under international law. This incentive is likely to persist so long as the EU fails to erect institutional mechanisms which secure joint responsibility for vulnerable migrants. (Sandven and Scherz 2022, p. 686)

Citing the example of the EU’s migration compacts with third countries such as Libya and Turkey, Sandven and Scherz argue that juridical recourse against the EU is utterly foreclosed because it ‘is currently able to shift the blame and to allege that these effects are due to the negligence of their collaborators since the legal documents that form the basis for their externalising techniques specify that all actors must respect human rights.’ (Sandven and Scherz 2022, p. 686) Accordingly, ‘European states can thus secure their borders by delegating tasks and competences to the EU level, whilst avoiding the responsibilities that otherwise would follow those tasks and competences—especially the obligation to accept and adjudicate asylum claims.’ (Sandven and Scherz 2022, p. 686) The upshot is that EU Member States along the Mediterranean littoral do not actually have to collapse into anarchy to evade their search and rescue operations. Rather, they can ‘manufacture’ the anarchy juridically – by outsourcing border policy enforcement to Frontex.

This has largely been borne out in the litigation so far. In *WS and others v Frontex*, the General Court dismissed a claim for damages by Syrian refugees against Frontex for expelling them from Greece in violation of the prohibition of refoulement; the EU could not be held directly responsible for the losses suffered because it lacked authority to assess the merits of return decisions of applications for international protection. (*WS and others v Frontex*, 2021) A subsequent application was initially dismissed with costs, not just for failing to provide sufficient evidence, but also because it ‘manifestly lacked any foundation in law.’ (*Hamoudi v Frontex*, 2023, para. 62) A legal practitioner remarked that the evidentiary burdens placed upon applicants – when all information about Frontex vessel movements and coordination with Member State officials – effectively amounted to a ‘“*probatio diabolica*”, an impossible proof…’ (Kunst 2025) In December 2025, the CJEU reversed, holding that in light of all probative evidence being in the possession of Frontex, the applicant could carry raise an inference of an illegal pushback through press reports and witness statements alone. (*Hamoudi v Frontex*, 2025)

Article 3(5) TEU – the very provision invoked in *Air Transport* to justify rights of extraterritorial jurisdiction over the high seas and airspace over third states – supplies the EU with another escape route more effective than unfair evidentiary burdens or restrictive standing rules. It was explicitly utilised as such in the *Western Sahara Campaign* decision, a preliminary reference from the United Kingdom, which questioned the validity of UK legislation implementing into the domestic legal order an international agreement between the EU and Morocco exploiting fisheries resources off the coast of the Western Sahara, a region widely considered to be illegally occupied by Morocco. (*Western Sahara Campaign*, 2018) The following passage merits close reading:

… with respect to the expression ‘waters falling within the sovereignty … of the Kingdom of Morocco’, used in… the Fisheries Partnership Agreement, it must be said that it would be contrary to the rules of international law referred to in … the present judgment, which the European Union must observe and which are applicable *mutatis mutandis* in this case, if it were agreed that the waters directly adjacent to the coast of the territory of Western Sahara were to be included within the scope of that agreement. Consequently, the European Union could not properly support any intention of the Kingdom of Morocco to include, by such means, the waters in question within the scope of that agreement. (*Western Sahara Campaign*, 2018, para. 71)

As a rule of interpretation barring the possibility of the EU be seen as having acted in violation of international law, Article 3(5) TEU had the salutary effect of prohibiting the application of the international agreement in waters off the coast of the Western Sahara, but only by excluding any accountability on the part of the EU for the negotiation and adoption of that treaty. Rather, any improper or untoward results of the EU’s knowing collusion with Morocco to exploit the fisheries resources of the illegally occupied Western Sahara were to be attributed entirely to Morocco.

The Kafkaesque implications of this rationale were demonstrated rather more starkly in slightly earlier *Polisario* litigation, which also concerned the validity of an EU-Morocco agreement to exploit the resources of Western Sahara. (*Polisario*, 2015; *Polisario*, 2016) There were, however, two differences. Firstly, the treaty in *Polisario* was for the exploitation of Sahrawi agricultural resources, rather than fisheries, which at first glance might appear to make it irrelevant to our current discussion about the sea. Not so: the General Court carefully avoided describing the Western Sahara as in occupied by Morocco, but instead described it as ‘disputed territory’ which it defined as ‘a territory in fact controlled by a non-member State, without the sovereignty of that State over that territory being recognised by the European Union and its Member States or, more generally, by all other States.’ (*Polisario*, 2015, para. 117) The concept of ‘disputed territory’ has no basis in international law, and might seem at first glance as a euphemism designed to prevent a diplomatic row with Morocco that would be engendered by calling it an ‘occupied territory’. The phrase, however, possesses a connotation ‘occupied territory’ lacks. ‘Occupied’ suggests land that definitively belongs to someone but which is for whatever reason in the possession of another. ‘Disputed’, by contrast, suggests that the question of who the land belongs to is undetermined, such that it is just as much a wilderness as the Grotian sea.

Second, *Polisario* was brought as an annulment proceeding rather than a preliminary reference. To summarise a technical discussion, preliminary references are effectively requests by Member State courts of the CJEU to elucidate questions of EU law in the *abstract*; the referring Member State court will then conduct its own proceedings consistently with the CJEU’s ‘answer.’ By contrast, applicants in annulment proceedings seek concrete review of EU actions; that is, a judicial determination that some act of the EU is *illegal*, and so, void. The EU law rules on standing to bring judicial review of acts of the EU have long been criticized as unduly onerous; in the case of ‘legislative’ acts, they effectively require the applicant to prove that the EU act operated as a ‘bill of attainder’ individually targeting her and her only. (Ganesh 2021 , pp 196–197)

In *Polisario*, however, the CJEU did not fundamentally make use of restrictive rules of evidence or procedure to deny standing to the applicant, which was the recognized national liberation movement of the Sahrawi people. Rather, without expressly identifying Article 3(5) TEU, it deployed it as an ‘Unrebuttable Presumption of Rectitude’ in favour of the EU: ‘in interpreting [the concerned treaty, EU Courts are] bound… to observe the rules of good faith interpretation… pursuant to which the interpretation of a treaty must be carried out by taking account of any relevant rules of international law applicable in the relations between the parties…’ The applicable rules of international law included the principle of self-determination, Article 29 VCLT on the territorial scope of treaties, and the relative effect of treaties. (*Polisario*, 2016, paras. 86–87) Because the application of the treaty to the territory of the Western Sahara would necessarily violate these rules, it followed that the operative term ‘territory of the Kingdom of Morocco’ in the treaty could not properly be interpreted as including the Western Sahara territory. Accordingly, per the CJEU’s logic, the treaty could not be of concern to the Front Polisario, which therefore meant it had no standing to seek judicial review.

Obviously, the question of whether the EU ‘could properly’ support Moroccan claims over Sahrawi waters differs from that of whether it *did* actually support them *improperly*. For the CJEU, however, the latter absolutely cannot be ‘conceded.’ (*Polisario*, 2016, para. 123) Accordingly, it did not matter in *Western Sahara Campaign* that the parties had literally drawn out their plans to fish in Sahrawi waters on a chart, (reproduced at *Opinion of A-General Wathelet*,* Western Sahara Campaign*, 2018, paras. 66–67) any more than it mattered in *Polisario* that the Commission sent officials from the Food and Veterinary Office to the Western Sahara at the invitation of Moroccan authorities to ensure agricultural produce met EU health standards. (*Polisario*, 2015, paras. 79–80; *Opinion of A-G Wathelet*,* Polisario*, 2016, paras. 98–99) These – and more – were all ‘too illegal to be true.’ (Kube 2017) Even if *Polisario* put an end to ongoing and future extraction of resources from the Western Sahara, the Article 3(5) TEU nevertheless insulates the EU from accountability for resources previously extracted under predecessor agreements *for decades*.

If the *Frontex* cases reveal how jurisdiction without accountability is produced by EU doctrines of the ‘pooling of sovereignty’ and conferral of competences, the Western Sahara cases, by contrast, reveal a more subtle stratagem: they show how *professions* of fidelity to EU law and human rights per Article 3(5) TEU can be instrumentalised. It is as if the EU acts in the fashion of a sovereign state when entering into the agreements with Morocco, but transubstantiates into an amorphous ‘union of values’ when called to account.

The aftermath of *Polisario* represents a third modality. Following the decision, the Council sought to overcome the hurdles imposed by the CJEU by, firstly, amending the agreements to refer expressly to the Western Sahara, and secondly, obtaining Sahrawi consent of some kind. The Front Polisario refused to participate in the renegotiation process out of concern it would amount to recognition of Moroccan control over the territory. (EU Commission 2018, p. 32) To get around this, the Commission and Council conducted extensive consultations in the Western Sahara resulting in a document that claimed, firstly, that EU market access and foreign direct investment would significantly advance the economic welfare of ‘local populations’ in the Western Sahara; and secondly, that ‘most people now living in Western Sahara [were] in favour of the extension of tariff preferences to products from Western Sahara under the EU-Morocco Association Agreement.’ (EU Commission 2018, p. 31) The emphasis on the benefits the Sahrawis would supposedly enjoy was presumably inspired by the Corell Memorandum, a legal opinion issued by the UN Under-Secretary-General for Legal Affairs and Legal Counsel, which urged ‘that the interests of the peoples of Non-Self-Governing Territories [were] paramount, and [that] their well-being and development is the “sacred trust” of their respective administering Powers…’ (Corell 2002, para. 22).

The Commission document leaves one with a distinct sense of human rights and development concerns being instrumentalized for resource extraction. As one commentator notes, it ‘examines the question almost entirely in economic terms’ as well as ‘downplayed the serious concern that the agreement would have the effect of cementing the *status quo* and consolidating Morocco’s illegal occupation of the territory.’ (Odermatt, 2021) This argument – that gains to general welfare can substitute for obligations of restitution for wrongfully taken resources – is of a piece with international negotiations on rights in Seabed resources where developed states contended that they would provide development aid rather than share mining revenues equitably under international supervision. (Enyew 2022, pp 491–492)

To its credit, the General Court resisted the Commission and other EU institutions, holding instead that the re-negotiated EU-Morocco fisheries and agricultural treaties were equally defective. Interestingly, it held that ‘the notion of “concerned populations” envisaged by the institutions’ differed fundamentally from that of the ‘people of the Western Sahara’, and, importantly, explained this difference not in terms of the fact that the current population of the Western Sahara includes vast numbers of Moroccans settled there illegally by the Moroccan government, but because the idea of a ‘local population’ lacks the ‘political content’ inherent in the concept of a ‘people’ deriving ‘notably, from the right of self-determination…’ (*Polisario I v Council*, 2021a, para. 329; *Polisario II*, 2021b, para. 337) The thought is that a ‘people’ is not a mere aggregation of individuals but a collective expressing itself only through public institutions that cannot be bypassed through local consultations.

The General Court also dismissed the EU institutions’ astonishingly impertinent claim that the Front Polisario’s entitlement to participate in international self-determination processes relating to the Western Sahara did not automatically translate to a right to participate in negotiations concerning the economic relations of that same territory, and, that in the absence of another UN-recognised organisation representing the Sahrawi people, requiring the Front Polisario’s participation would effectively give it a ‘right of veto’. The institutions appear to have supposed the EU to possess a vested option in Sahrawi resources, upon which the Front Polisario’s non-cooperation constituted an unreasonable constraint. The General Court’s response was pleasingly curt:

It suffices to recall… [that] it was not for the Council to decide whether it was possible to do without the consent of the people of the Western Sahara to conclude the disputed agreement.’ (*Polisario I*, 2021a, para. 346; *Polisario II*, 2021b, para. 364).

On appeal, the CJEU upheld the General Court’s decisions, but on a reasoning no less spurious than that in the earlier Western Sahara cases. Beginning with the contention that customary international law presumes the consent of third parties to a treaty conferring *rights* upon them, the CJEU proceeded to find that the EU institutions were not required to obtain their ‘express’ consent, because neither the fisheries nor the agricultural exploitation treaties imposed *obligations* on the Sahrawi people. Instead, the EU institutions could ‘presume’ their consent, as long as it the Sahrawi people received ‘a specific, tangible, substantial and verifiable benefit from the exploitation of that territory’s natural resources which is proportional to the degree of that exploitation,’ alongside ‘guarantees’ that such exploitation would be sustainable, and ‘control mechanisms’ to ensure that the Sahrawis actually obtained that benefit. (*Polisario I*, 2024, paras. 180–181; *Polisario II*, 2024, paras. 152–153) It was only the failure of the institutions to demonstrate these that was fatal to their case.

In contrast to the relative republicanism of the General Court, the CJEU’s attitude to Sahrawis and the people of other NSGTs is deeply reminiscent of several strategies used by proponents of colonialism. As a recent commentator observes, these recent CJEU’s decisions are singular in that they ‘introduce a quite novel concept of presumed consent by the people of NSGTs as a third party to international agreements.’ (Szydło 2025, p. 8) In the deployment of Article 3(5) TEU as an Unrebuttable Presumption of Rectitude, the notion that the exploitation of an NSGT’s resources is not a burden but a positive blessing to its people, one can see clear shades of the inexhaustible Grotian commons, and of the unshakeable faith of Earl Grey and Leopold in their civilising missions in a fallen wider world.

## Conclusion

The foregoing paragraphs elucidated some modalities by which the EU’s invocation of human rights in justification of extraterritorial legislation perpetuates rather than remedies the juridical vacuum that has plagued the oceans, skies, and other wildernesses since Grotius. By tracing the genealogy from Grotius’s defence of private colonial warfare to contemporary EU practices, we see how the basic conceptual architecture of maritime law – premised on the seas either as institutional wilderness or as former wildernesses actively enclosed at identifiable points in history – enables rather than constrains the very abuses it purports to address. Crucially, the EU’s structural ambiguity between international legal person and normative union, combined with its restrictive judicial review mechanisms, allows it to exploit these Grotian ‘black holes’ whilst at the same time cloaking neo-imperial resource extraction in the language of universal values. This is not a departure from, but the logical culmination of a legal framework that, despite rejecting Grotius’s assumptions about inexhaustibility and non-enclosure, remains trapped within his basic categories. Only by abandoning them, and conceiving the seas as *inherently* institutional global public goods, can international law end such forms of legitimized domination on the high seas and other contested spaces.

## Case List

Case C-104/16 P Council v Front Polisario, 2016, ECLI: EU: C:2016:973.

Case C‑266/16 Western Sahara Campaign UK, The Queen v Commissioners for Her Majesty’s Revenue and Customs, Secretary of State for Environment, Food and Rural Affairs, 2018, ECLI: EU: C:2018:118.

Case C‑286/90 Anklagemyndigheden v Poulsen and Diva Navigation, 1992, ECLI:EU:C:1992:453. 

Case C-347/10, Salemink, 2012, EU: C:2012:17.

Case C-366/10, Air Transport Association of America and ors v Secretary of State for Energy and Climate Change, 2011, ECLI:EU:C:2011:864.

Case T-136/22, Hamoudi v European Border and Coast Guard Agency (Frontex), 2023, ECLI: EU: T:2023:821.

Case C-136/24 P, Hamoudi v European Border and Coast Guard Agency, 2025, ECLI: EU: C:2025:977.

Case T-279/19, Front Polisario v Council, 2021, ECLI: EU: T:2021:639.

Case T-512/12, Front Polisario v Council, 2015, ECLI:EU:T:2015:953.

Case T-600/21, WS and others v Frontex, 2021, ECLI:EU:T:2023:492.

Fisheries Jurisdiction Case, 1974, ICJ 3.

Fur Seal Arbitration (1895) Proceedings of the Tribunal of Arbitration, Convened at Paris, Under the Treaty Between the United States of America and Great Britain, Concluded at Washington, February 20, 1892, for the Determination of Questions Between the Two Governments Concerning the Jurisdictional Rights of the United States in the Waters of Bering Sea. U.S. Government Printing Office.

Joined Cases 89/85, 104/85, 114/85, 116/85, 117/85 & 125/85 to 129/85 Ahlström Osakeyhtiö and others v Commission (*Wood Pulp I*), 1988, ECLI:EU:C:1993:120. 

Joined Cases C-778/21 P and C-798/21 P European Commission v Front Polisario and Council of the European Union v Front Polisario, 2024, ECLI: EU: C:2024:833.

Joined Cases C-779/21 P and C-799/21 P European Commission v Front Polisario and Council of the European Union v Front Polisario, 2024, ECLI: EU: C:2024:835.

Joined Cases T-344/19 and T-356/19, Front Polisario v Council, 2021, ECLI: EU: T:2021:640.

North Sea Continental Shelf Cases, 1969, ICJ 3.

Opinion of Advocate General Cruz Villalón, Case C-347/10, Salemink, 2011a, EU: C:2011:562.

Opinion of Advocate General Darmon, Case 89/85, 104/85, 114/85, 116/85, 117/85 and 125/85 to 129/85 Ahlström Osakeyhtiö and others v Commission (*Wood Pulp* *I*), 1988, EU: C:1988:258.

Opinion of Advocate General Kokott, Case C-366/10, Air Transport Association of America and ors v Secretary of State for Energy and Climate Change, 2011, EU: C:2011:637.

Opinion of Advocate General Wathelet, Case C-104/16 P, Council v Front Polisario, 2016, ECLI: EU: C:2016:677.

Opinion of Advocate General Wathelet, Case C‑266/16, Western Sahara Campaign UK, The Queen v Commissioners for Her Majesty’s Revenue and Customs, Secretary of State for Environment, Food and Rural Affairs, 2018, ECLI: EU: C:2018:1.

SS and others v Italy, 2025, ECLI:CE:ECHR:2025:0520DEC002166018.

The Apollon, 1824, 22 US (9 Wheat) 362.

## Data Availability

No datasets were generated or analysed during the current study.

## Footnotes

1 Institutionless, not lawless. There is law that governs on the seas: natural law.
